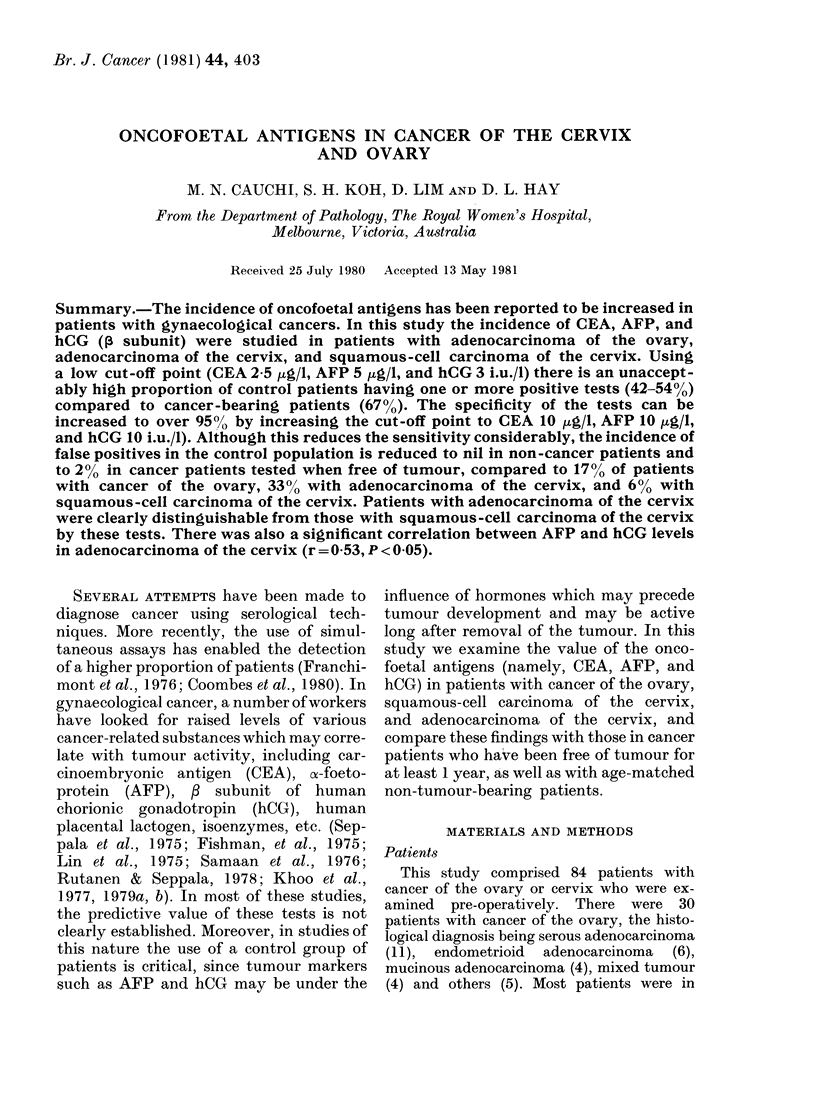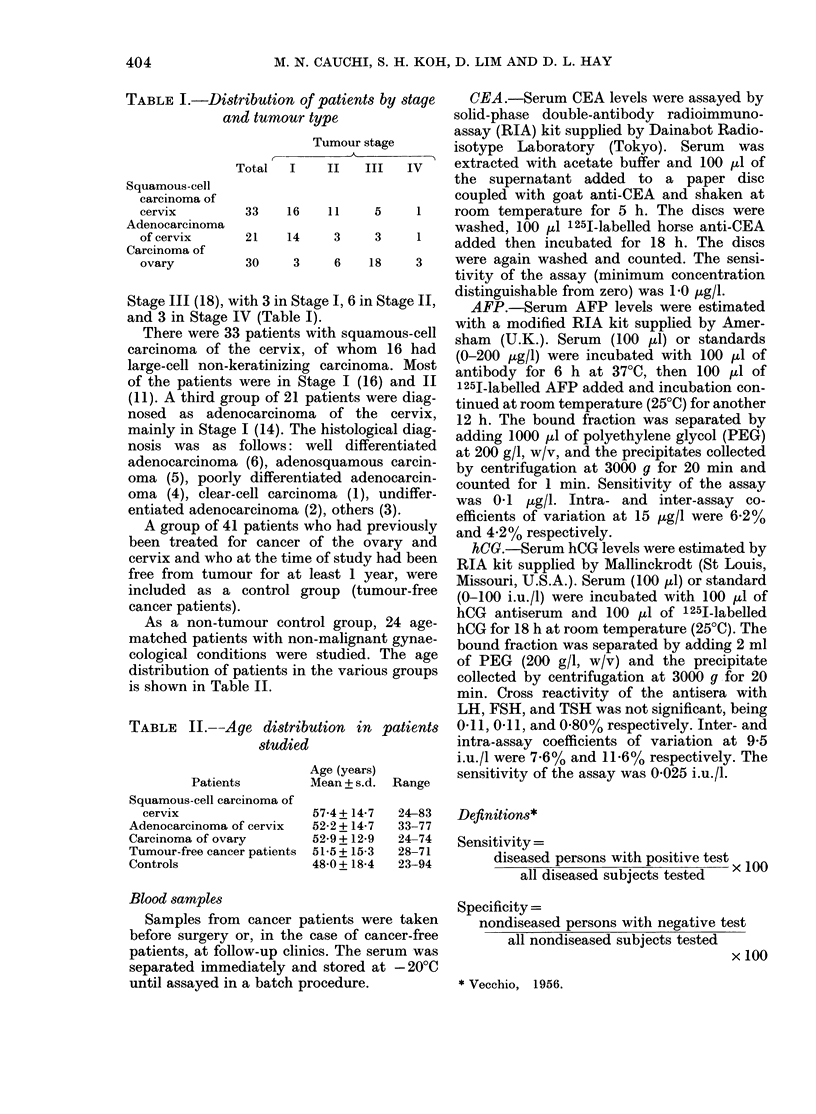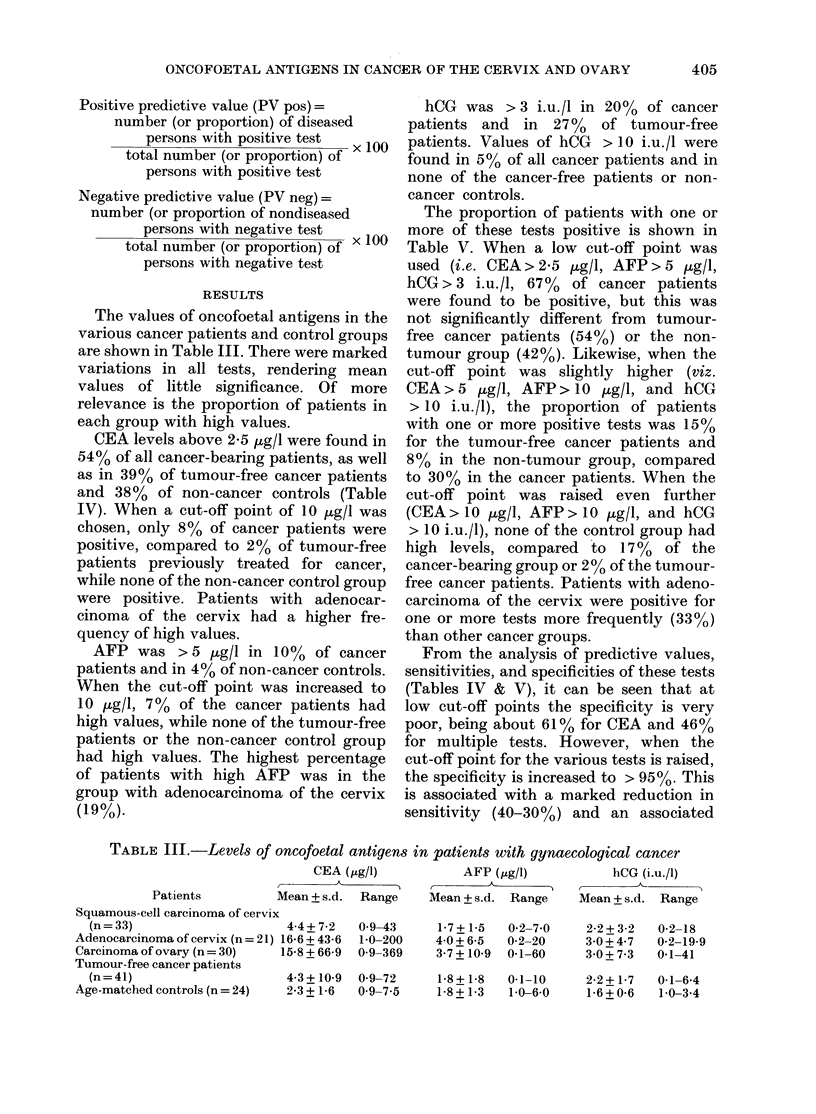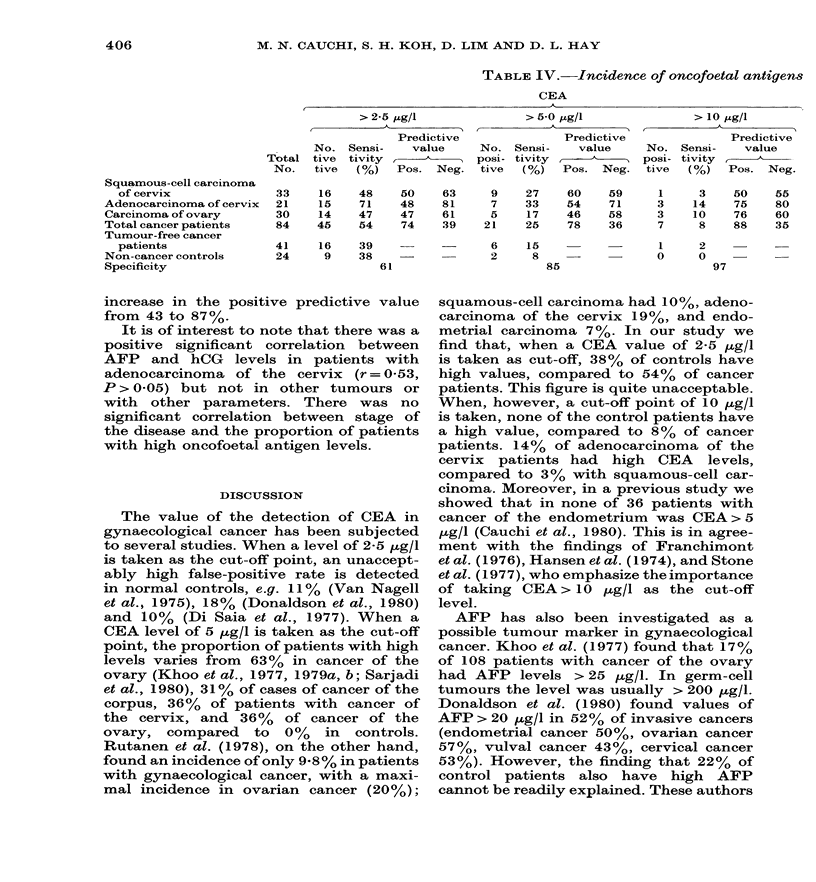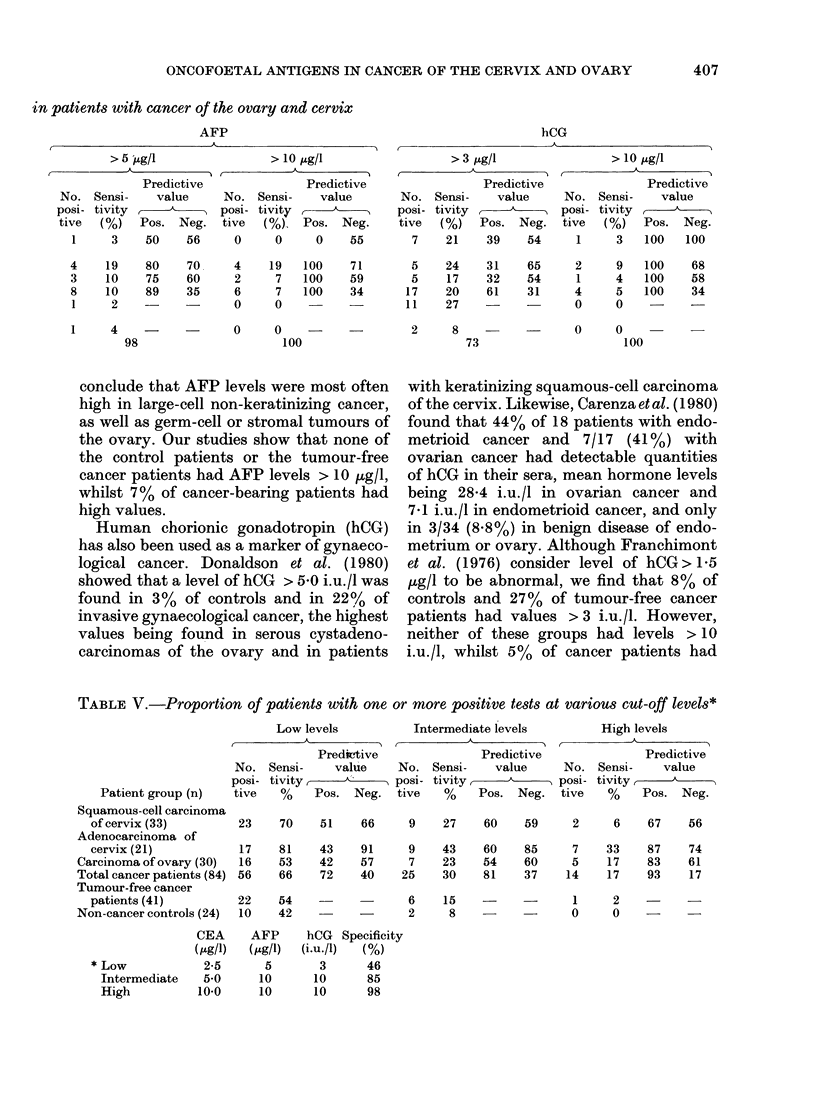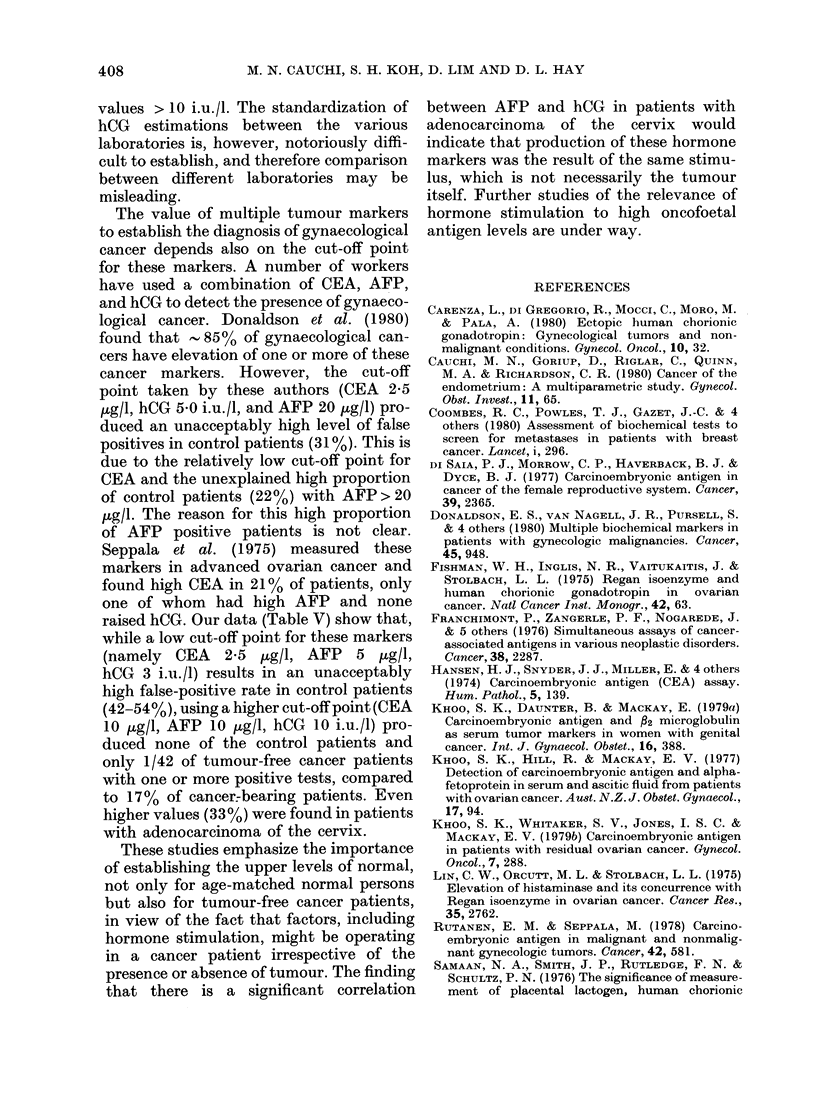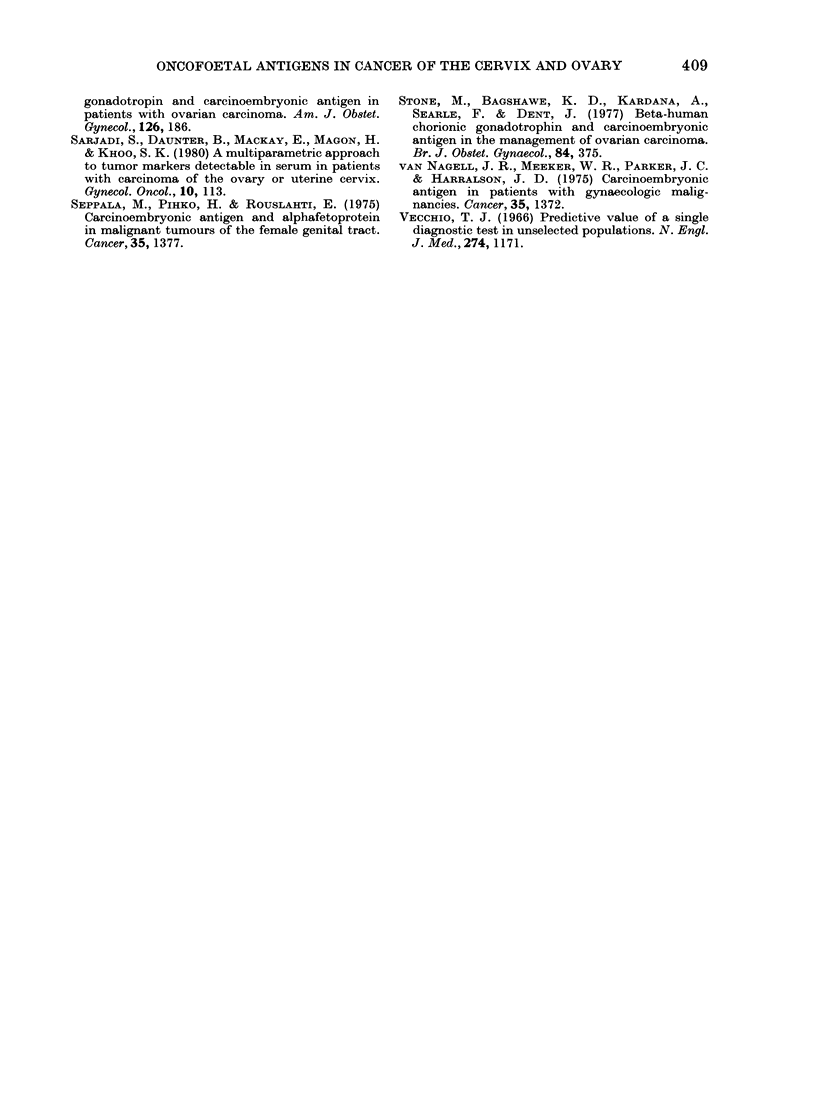# Oncofoetal antigens in cancer of the cervix and ovary.

**DOI:** 10.1038/bjc.1981.198

**Published:** 1981-09

**Authors:** M. N. Cauchi, S. H. Koh, D. Lim, D. L. Hay

## Abstract

The incidence of oncofoetal antigens has been reported to be increased in patients with gynaecological cancers. In this study the incidence of CEA, AFP, and hCG (beta subunit) were studied in patients with adenocarcinoma of the ovary, adenocarcinoma of the cervix, and squamous-cell carcinoma of the cervix. Using a low cut-off point (CEA 2.5 microgram/l, AFP 5 microgram/l, and hCG 3 i.u./l) there is an unacceptably high proportion of control patients having one or more positive tests (42-54%) compared to cancer-bearing patients (67%). The specificity of the tests can be increased to over 95% by increasing the cut-off point to CEA 10 microgram/l, AFP 10 microgram/l, and hCG 10 i.u./l). Although this reduces the sensitivity considerably, the incidence of false positives in the control population is reduced to nil in non-cancer patients and to 2% in cancer patients tested when free of tumour, compared to 17% of patients with cancer of the ovary, 33% with adenocarcinoma of the cervix, and 6% with squamous-cell carcinoma of the cervix. Patients with adenocarcinoma of the cervix were clearly distinguishable from those with squamous-cell carcinoma of the cervix by these tests. There was also a significant correlation between AFP and hCG levels in adenocarcinoma of the cervix (r = 0.53, P less than 0.05).


*Br. J. Cancer* (1981) **44**, 403

### ONCOFOETAL ANTIGENS IN CANCER OF THE CERVIX AND OVARY

M. N. CAUCHI, S. H. KOH, D. LIM AND D. L. HAY

*From the Department of Pathology, The Royal Women's Hospital, Melbourne, Victoria, Australia*

Received 25 July 1980 Accepted 13 May 1981

**Summary.—The incidence of oncofoetal antigens has been reported to be increased in patients with gynaecological cancers. In this study the incidence of CEA, AFP, and hCG (β subunit) were studied in patients with adenocarcinoma of the ovary, adenocarcinoma of the cervix, and squamous-cell carcinoma of the cervix. Using a low cut-off point (CEA 2·5 μg/l, AFP 5 μg/l, and hCG 3 i.u./l) there is an unacceptably high proportion of control patients having one or more positive tests (42–54%) compared to cancer-bearing patients (67%). The specificity of the tests can be increased to over 95% by increasing the cut-off point to CEA 10 μg/l, AFP 10 μg/l, and hCG 10 i.u./l). Although this reduces the sensitivity considerably, the incidence of false positives in the control population is reduced to nil in non-cancer patients and to 2% in cancer patients tested when free of tumour, compared to 17% of patients with cancer of the ovary, 33% with adenocarcinoma of the cervix, and 6% with squamous-cell carcinoma of the cervix. Patients with adenocarcinoma of the cervix were clearly distinguishable from those with squamous-cell carcinoma of the cervix by these tests. There was also a significant correlation between AFP and hCG levels in adenocarcinoma of the cervix ($r = 0{\cdot}53, P < 0{\cdot}05$).**

SEVERAL ATTEMPTS have been made to diagnose cancer using serological techniques. More recently, the use of simultaneous assays has enabled the detection of a higher proportion of patients (Franchimont *et al.*, 1976; Coombes *et al.*, 1980). In gynaecological cancer, a number of workers have looked for raised levels of various cancer-related substances which may correlate with tumour activity, including carcinoembryonic antigen (CEA), α-foetoprotein (AFP), β subunit of human chorionic gonadotropin (hCG), human placental lactogen, isoenzymes, etc. (Seppala *et al.*, 1975; Fishman, *et al.*, 1975; Lin *et al.*, 1975; Samaan *et al.*, 1976; Rutanen & Seppala, 1978; Khoo *et al.*, 1977, 1979*a*, *b*). In most of these studies, the predictive value of these tests is not clearly established. Moreover, in studies of this nature the use of a control group of patients is critical, since tumour markers such as AFP and hCG may be under the influence of hormones which may precede tumour development and may be active long after removal of the tumour. In this study we examine the value of the oncofoetal antigens (namely, CEA, AFP, and hCG) in patients with cancer of the ovary, squamous-cell carcinoma of the cervix, and adenocarcinoma of the cervix, and compare these findings with those in cancer patients who have been free of tumour for at least 1 year, as well as with age-matched non-tumour-bearing patients.

### MATERIALS AND METHODS

### *Patients*

This study comprised 84 patients with cancer of the ovary or cervix who were examined pre-operatively. There were 30 patients with cancer of the ovary, the histological diagnosis being serous adenocarcinoma (11), endometrioid adenocarcinoma (6), mucinous adenocarcinoma (4), mixed tumour (4) and others (5). Most patients were in

TABLE I.—*Distribution of patients by stage and tumour type*

| | | Tumour stage | | | |
|---|---|---|---|---|---|
| | Total | I | II | III | IV |
| Squamous-cell carcinoma of cervix | 33 | 16 | 11 | 5 | 1 |
| Adenocarcinoma of cervix | 21 | 14 | 3 | 3 | 1 |
| Carcinoma of ovary | 30 | 3 | 6 | 18 | 3 |

Stage III (18), with 3 in Stage I, 6 in Stage II, and 3 in Stage IV (Table I).

There were 33 patients with squamous-cell carcinoma of the cervix, of whom 16 had large-cell non-keratinizing carcinoma. Most of the patients were in Stage I (16) and II (11). A third group of 21 patients were diagnosed as adenocarcinoma of the cervix, mainly in Stage I (14). The histological diagnosis was as follows: well differentiated adenocarcinoma (6), adenosquamous carcinoma (5), poorly differentiated adenocarcinoma (4), clear-cell carcinoma (1), undifferentiated adenocarcinoma (2), others (3).

A group of 41 patients who had previously been treated for cancer of the ovary and cervix and who at the time of study had been free from tumour for at least 1 year, were included as a control group (tumour-free cancer patients).

As a non-tumour control group, 24 age-matched patients with non-malignant gynaecological conditions were studied. The age distribution of patients in the various groups is shown in Table II.

TABLE II.—*Age distribution in patients studied*

| Patients | Age (years) Mean ± s.d. | Range |
|---|---|---|
| Squamous-cell carcinoma of cervix | 57·4 ± 14·7 | 24–83 |
| Adenocarcinoma of cervix | 52·2 ± 14·7 | 33–77 |
| Carcinoma of ovary | 52·9 ± 12·9 | 24–74 |
| Tumour-free cancer patients | 51·5 ± 15·3 | 28–71 |
| Controls | 48·0 ± 18·4 | 23–94 |

*Blood samples*

Samples from cancer patients were taken before surgery or, in the case of cancer-free patients, at follow-up clinics. The serum was separated immediately and stored at −20°C until assayed in a batch procedure.

*CEA*.—Serum CEA levels were assayed by solid-phase double-antibody radioimmunoassay (RIA) kit supplied by Dainabot Radioisotype Laboratory (Tokyo). Serum was extracted with acetate buffer and 100 μl of the supernatant added to a paper disc coupled with goat anti-CEA and shaken at room temperature for 5 h. The discs were washed, 100 μl $^{125}$I-labelled horse anti-CEA added then incubated for 18 h. The discs were again washed and counted. The sensitivity of the assay (minimum concentration distinguishable from zero) was 1·0 μg/l.

*AFP*.—Serum AFP levels were estimated with a modified RIA kit supplied by Amersham (U.K.). Serum (100 μl) or standards (0–200 μg/l) were incubated with 100 μl of antibody for 6 h at 37°C, then 100 μl of $^{125}$I-labelled AFP added and incubation continued at room temperature (25°C) for another 12 h. The bound fraction was separated by adding 1000 μl of polyethylene glycol (PEG) at 200 g/l, w/v, and the precipitates collected by centrifugation at 3000 *g* for 20 min and counted for 1 min. Sensitivity of the assay was 0·1 μg/l. Intra- and inter-assay coefficients of variation at 15 μg/l were 6·2% and 4·2% respectively.

*hCG*.—Serum hCG levels were estimated by RIA kit supplied by Mallinckrodt (St Louis, Missouri, U.S.A.). Serum (100 μl) or standard (0–100 i.u./l) were incubated with 100 μl of hCG antiserum and 100 μl of $^{125}$I-labelled hCG for 18 h at room temperature (25°C). The bound fraction was separated by adding 2 ml of PEG (200 g/l, w/v) and the precipitate collected by centrifugation at 3000 *g* for 20 min. Cross reactivity of the antisera with LH, FSH, and TSH was not significant, being 0·11, 0·11, and 0·80% respectively. Inter- and intra-assay coefficients of variation at 9·5 i.u./l were 7·6% and 11·6% respectively. The sensitivity of the assay was 0·025 i.u./l.

*Definitions**

$$\text{Sensitivity} = \frac{\text{diseased persons with positive test}}{\text{all diseased subjects tested}} \times 100$$

$$\text{Specificity} = \frac{\text{nondiseased persons with negative test}}{\text{all nondiseased subjects tested}} \times 100$$

* Vecchio, 1956.

Positive predictive value (PV pos) =

$$\frac{\text{number (or proportion) of diseased persons with positive test}}{\text{total number (or proportion) of persons with positive test}} \times 100$$

Negative predictive value (PV neg) =

$$\frac{\text{number (or proportion of nondiseased persons with negative test}}{\text{total number (or proportion) of persons with negative test}} \times 100$$

RESULTS

The values of oncofoetal antigens in the various cancer patients and control groups are shown in Table III. There were marked variations in all tests, rendering mean values of little significance. Of more relevance is the proportion of patients in each group with high values.

CEA levels above 2·5 μg/l were found in 54% of all cancer-bearing patients, as well as in 39% of tumour-free cancer patients and 38% of non-cancer controls (Table IV). When a cut-off point of 10 μg/l was chosen, only 8% of cancer patients were positive, compared to 2% of tumour-free patients previously treated for cancer, while none of the non-cancer control group were positive. Patients with adenocarcinoma of the cervix had a higher frequency of high values.

AFP was > 5 μg/l in 10% of cancer patients and in 4% of non-cancer controls. When the cut-off point was increased to 10 μg/l, 7% of the cancer patients had high values, while none of the tumour-free patients or the non-cancer control group had high values. The highest percentage of patients with high AFP was in the group with adenocarcinoma of the cervix (19%).

hCG was > 3 i.u./l in 20% of cancer patients and in 27% of tumour-free patients. Values of hCG > 10 i.u./l were found in 5% of all cancer patients and in none of the cancer-free patients or non-cancer controls.

The proportion of patients with one or more of these tests positive is shown in Table V. When a low cut-off point was used (*i.e.* CEA > 2·5 μg/l, AFP > 5 μg/l, hCG > 3 i.u./l, 67% of cancer patients were found to be positive, but this was not significantly different from tumour-free cancer patients (54%) or the non-tumour group (42%). Likewise, when the cut-off point was slightly higher (*viz.* CEA > 5 μg/l, AFP > 10 μg/l, and hCG > 10 i.u./l), the proportion of patients with one or more positive tests was 15% for the tumour-free cancer patients and 8% in the non-tumour group, compared to 30% in the cancer patients. When the cut-off point was raised even further (CEA > 10 μg/l, AFP > 10 μg/l, and hCG > 10 i.u./l), none of the control group had high levels, compared to 17% of the cancer-bearing group or 2% of the tumour-free cancer patients. Patients with adenocarcinoma of the cervix were positive for one or more tests more frequently (33%) than other cancer groups.

From the analysis of predictive values, sensitivities, and specificities of these tests (Tables IV & V), it can be seen that at low cut-off points the specificity is very poor, being about 61% for CEA and 46% for multiple tests. However, when the cut-off point for the various tests is raised, the specificity is increased to > 95%. This is associated with a marked reduction in sensitivity (40–30%) and an associated

TABLE III.—*Levels of oncofoetal antigens in patients with gynaecological cancer*

| Patients | CEA (μg/l) Mean ± s.d. | CEA (μg/l) Range | AFP (μg/l) Mean ± s.d. | AFP (μg/l) Range | hCG (i.u./l) Mean ± s.d. | hCG (i.u./l) Range |
|---|---|---|---|---|---|---|
| Squamous-cell carcinoma of cervix (n = 33) | 4·4 ± 7·2 | 0·9–43 | 1·7 ± 1·5 | 0·2–7·0 | 2·2 ± 3·2 | 0·2–18 |
| Adenocarcinoma of cervix (n = 21) | 16·6 ± 43·6 | 1·0–200 | 4·0 ± 6·5 | 0·2–20 | 3·0 ± 4·7 | 0·2–19·9 |
| Carcinoma of ovary (n = 30) | 15·8 ± 66·9 | 0·9–369 | 3·7 ± 10·9 | 0·1–60 | 3·0 ± 7·3 | 0·1–41 |
| Tumour-free cancer patients (n = 41) | 4·3 ± 10·9 | 0·9–72 | 1·8 ± 1·8 | 0·1–10 | 2·2 ± 1·7 | 0·1–6·4 |
| Age-matched controls (n = 24) | 2·3 ± 1·6 | 0·9–7·5 | 1·8 ± 1·3 | 1·0–6·0 | 1·6 ± 0·6 | 1·0–3·4 |

TABLE IV.—*Incidence of oncofoetal antigens*

| | | CEA | | | | | | | | | | | |
|---|---|---|---|---|---|---|---|---|---|---|---|---|---|
| | | > 2·5 μg/l | | | | > 5·0 μg/l | | | | > 10 μg/l | | | |
| | Total No. | No. tive tive | Sensitivity (%) | Predictive value Pos. | Predictive value Neg. | No. positive | Sensitivity (%) | Predictive value Pos. | Predictive value Neg. | No. positive | Sensitivity (%) | Predictive value Pos. | Predictive value Neg. |
| Squamous-cell carcinoma of cervix | 33 | 16 | 48 | 50 | 63 | 9 | 27 | 60 | 59 | 1 | 3 | 50 | 55 |
| Adenocarcinoma of cervix | 21 | 15 | 71 | 48 | 81 | 7 | 33 | 54 | 71 | 3 | 14 | 75 | 80 |
| Carcinoma of ovary | 30 | 14 | 47 | 47 | 61 | 5 | 17 | 46 | 58 | 3 | 10 | 76 | 60 |
| Total cancer patients | 84 | 45 | 54 | 74 | 39 | 21 | 25 | 78 | 36 | 7 | 8 | 88 | 35 |
| Tumour-free cancer patients | 41 | 16 | 39 | — | — | 6 | 15 | — | — | 1 | 2 | — | — |
| Non-cancer controls | 24 | 9 | 38 | — | — | 2 | 8 | — | — | 0 | 0 | — | — |
| Specificity | | | | 61 | | | | 85 | | | | 97 | |

increase in the positive predictive value from 43 to 87%.

It is of interest to note that there was a positive significant correlation between AFP and hCG levels in patients with adenocarcinoma of the cervix ($r = 0{\cdot}53$, $P > 0{\cdot}05$) but not in other tumours or with other parameters. There was no significant correlation between stage of the disease and the proportion of patients with high oncofoetal antigen levels.

### DISCUSSION

The value of the detection of CEA in gynaecological cancer has been subjected to several studies. When a level of 2·5 μg/l is taken as the cut-off point, an unacceptably high false-positive rate is detected in normal controls, *e.g.* 11% (Van Nagell *et al.*, 1975), 18% (Donaldson *et al.*, 1980) and 10% (Di Saia *et al.*, 1977). When a CEA level of 5 μg/l is taken as the cut-off point, the proportion of patients with high levels varies from 63% in cancer of the ovary (Khoo *et al.*, 1977, 1979*a*, *b*; Sarjadi *et al.*, 1980), 31% of cases of cancer of the corpus, 36% of patients with cancer of the cervix, and 36% of cancer of the ovary, compared to 0% in controls. Rutanen *et al.* (1978), on the other hand, found an incidence of only 9·8% in patients with gynaecological cancer, with a maximal incidence in ovarian cancer (20%); squamous-cell carcinoma had 10%, adenocarcinoma of the cervix 19%, and endometrial carcinoma 7%. In our study we find that, when a CEA value of 2·5 μg/l is taken as cut-off, 38% of controls have high values, compared to 54% of cancer patients. This figure is quite unacceptable. When, however, a cut-off point of 10 μg/l is taken, none of the control patients have a high value, compared to 8% of cancer patients. 14% of adenocarcinoma of the cervix patients had high CEA levels, compared to 3% with squamous-cell carcinoma. Moreover, in a previous study we showed that in none of 36 patients with cancer of the endometrium was CEA > 5 μg/l (Cauchi *et al.*, 1980). This is in agreement with the findings of Franchimont *et al.* (1976), Hansen *et al.* (1974), and Stone *et al.* (1977), who emphasize the importance of taking CEA > 10 μg/l as the cut-off level.

AFP has also been investigated as a possible tumour marker in gynaecological cancer. Khoo *et al.* (1977) found that 17% of 108 patients with cancer of the ovary had AFP levels > 25 μg/l. In germ-cell tumours the level was usually > 200 μg/l. Donaldson *et al.* (1980) found values of AFP > 20 μg/l in 52% of invasive cancers (endometrial cancer 50%, ovarian cancer 57%, vulval cancer 43%, cervical cancer 53%). However, the finding that 22% of control patients also have high AFP cannot be readily explained. These authors

*in patients with cancer of the ovary and cervix*

| AFP > 5 µg/l No. positive | Sensitivity (%) | Predictive value Pos. | Neg. | AFP > 10 µg/l No. positive | Sensitivity (%) | Predictive value Pos. | Neg. | hCG > 3 µg/l No. positive | Sensitivity (%) | Predictive value Pos. | Neg. | hCG > 10 µg/l No. positive | Sensitivity (%) | Predictive value Pos. | Neg. |
|---|---|---|---|---|---|---|---|---|---|---|---|---|---|---|---|
| 1 | 3 | 50 | 56 | 0 | 0 | 0 | 55 | 7 | 21 | 39 | 54 | 1 | 3 | 100 | 100 |
| 4 | 19 | 80 | 70 | 4 | 19 | 100 | 71 | 5 | 24 | 31 | 65 | 2 | 9 | 100 | 68 |
| 3 | 10 | 75 | 60 | 2 | 7 | 100 | 59 | 5 | 17 | 32 | 54 | 1 | 4 | 100 | 58 |
| 8 | 10 | 89 | 35 | 6 | 7 | 100 | 34 | 17 | 20 | 61 | 31 | 4 | 5 | 100 | 34 |
| 1 | 2 | — | — | 0 | 0 | — | — | 11 | 27 | — | — | 0 | 0 | — | — |
| 1 | 4 | — | — | 0 | 0 | — | — | 2 | 8 | — | — | 0 | 0 | — | — |
| | 98 | | | | 100 | | | | 73 | | | | 100 | | |

conclude that AFP levels were most often high in large-cell non-keratinizing cancer, as well as germ-cell or stromal tumours of the ovary. Our studies show that none of the control patients or the tumour-free cancer patients had AFP levels > 10 µg/l, whilst 7% of cancer-bearing patients had high values.

Human chorionic gonadotropin (hCG) has also been used as a marker of gynaecological cancer. Donaldson *et al.* (1980) showed that a level of hCG > 5·0 i.u./l was found in 3% of controls and in 22% of invasive gynaecological cancer, the highest values being found in serous cystadenocarcinomas of the ovary and in patients with keratinizing squamous-cell carcinoma of the cervix. Likewise, Carenza *et al.* (1980) found that 44% of 18 patients with endometrioid cancer and 7/17 (41%) with ovarian cancer had detectable quantities of hCG in their sera, mean hormone levels being 28·4 i.u./l in ovarian cancer and 7·1 i.u./l in endometrioid cancer, and only in 3/34 (8·8%) in benign disease of endometrium or ovary. Although Franchimont *et al.* (1976) consider level of hCG > 1·5 µg/l to be abnormal, we find that 8% of controls and 27% of tumour-free cancer patients had values > 3 i.u./l. However, neither of these groups had levels > 10 i.u./l, whilst 5% of cancer patients had

TABLE V.—*Proportion of patients with one or more positive tests at various cut-off levels**

| Patient group (n) | Low levels No. positive | Sensitivity % | Predictive value Pos. | Neg. | Intermediate levels No. positive | Sensitivity % | Predictive value Pos. | Neg. | High levels No. positive | Sensitivity % | Predictive value Pos. | Neg. |
|---|---|---|---|---|---|---|---|---|---|---|---|---|
| Squamous-cell carcinoma of cervix (33) | 23 | 70 | 51 | 66 | 9 | 27 | 60 | 59 | 2 | 6 | 67 | 56 |
| Adenocarcinoma of cervix (21) | 17 | 81 | 43 | 91 | 9 | 43 | 60 | 85 | 7 | 33 | 87 | 74 |
| Carcinoma of ovary (30) | 16 | 53 | 42 | 57 | 7 | 23 | 54 | 60 | 5 | 17 | 83 | 61 |
| Total cancer patients (84) | 56 | 66 | 72 | 40 | 25 | 30 | 81 | 37 | 14 | 17 | 93 | 17 |
| Tumour-free cancer patients (41) | 22 | 54 | — | — | 6 | 15 | — | — | 1 | 2 | — | — |
| Non-cancer controls (24) | 10 | 42 | — | — | 2 | 8 | — | — | 0 | 0 | — | — |

| | CEA (µg/l) | AFP (µg/l) | hCG (i.u./l) | Specificity (%) |
|---|---|---|---|---|
| * Low | 2·5 | 5 | 3 | 46 |
| Intermediate | 5·0 | 10 | 10 | 85 |
| High | 10·0 | 10 | 10 | 98 |

values > 10 i.u./l. The standardization of hCG estimations between the various laboratories is, however, notoriously difficult to establish, and therefore comparison between different laboratories may be misleading.

The value of multiple tumour markers to establish the diagnosis of gynaecological cancer depends also on the cut-off point for these markers. A number of workers have used a combination of CEA, AFP, and hCG to detect the presence of gynaecological cancer. Donaldson *et al.* (1980) found that ~85% of gynaecological cancers have elevation of one or more of these cancer markers. However, the cut-off point taken by these authors (CEA 2·5 μg/l, hCG 5·0 i.u./l, and AFP 20 μg/l) produced an unacceptably high level of false positives in control patients (31%). This is due to the relatively low cut-off point for CEA and the unexplained high proportion of control patients (22%) with AFP > 20 μg/l. The reason for this high proportion of AFP positive patients is not clear. Seppala *et al.* (1975) measured these markers in advanced ovarian cancer and found high CEA in 21% of patients, only one of whom had high AFP and none raised hCG. Our data (Table V) show that, while a low cut-off point for these markers (namely CEA 2·5 μg/l, AFP 5 μg/l, hCG 3 i.u./l) results in an unacceptably high false-positive rate in control patients (42–54%), using a higher cut-off point (CEA 10 μg/l, AFP 10 μg/l, hCG 10 i.u./l) produced none of the control patients and only 1/42 of tumour-free cancer patients with one or more positive tests, compared to 17% of cancer-bearing patients. Even higher values (33%) were found in patients with adenocarcinoma of the cervix.

These studies emphasize the importance of establishing the upper levels of normal, not only for age-matched normal persons but also for tumour-free cancer patients, in view of the fact that factors, including hormone stimulation, might be operating in a cancer patient irrespective of the presence or absence of tumour. The finding that there is a significant correlation between AFP and hCG in patients with adenocarcinoma of the cervix would indicate that production of these hormone markers was the result of the same stimulus, which is not necessarily the tumour itself. Further studies of the relevance of hormone stimulation to high oncofoetal antigen levels are under way.

### REFERENCES

CARENZA, L., DI GREGORIO, R., MOCCI, C., MORO, M. & PALA, A. (1980) Ectopic human chorionic gonadotropin: Gynecological tumors and non-malignant conditions. *Gynecol. Oncol.*, **10**, 32.

CAUCHI, M. N., GORIUP, D., RIGLAR, C., QUINN, M. A. & RICHARDSON, C. R. (1980) Cancer of the endometrium: A multiparametric study. *Gynecol. Obst. Invest.*, **11**, 65.

COOMBES, R. C., POWLES, T. J., GAZET, J.-C. & 4 others (1980) Assessment of biochemical tests to screen for metastases in patients with breast cancer. *Lancet*, i, 296.

DI SAIA, P. J., MORROW, C. P., HAVERBACK, B. J. & DYCE, B. J. (1977) Carcinoembryonic antigen in cancer of the female reproductive system. *Cancer*, **39**, 2365.

DONALDSON, E. S., VAN NAGELL, J. R., PURSELL, S. & 4 others (1980) Multiple biochemical markers in patients with gynecologic malignancies. *Cancer*, **45**, 948.

FISHMAN, W. H., INGLIS, N. R., VAITUKAITIS, J. & STOLBACH, L. L. (1975) Regan isoenzyme and human chorionic gonadotropin in ovarian cancer. *Natl Cancer Inst. Monogr.*, **42**, 63.

FRANCHIMONT, P., ZANGERLE, P. F., NOGAREDE, J. & 5 others (1976) Simultaneous assays of cancer-associated antigens in various neoplastic disorders. *Cancer*, **38**, 2287.

HANSEN, H. J., SNYDER, J. J., MILLER, E. & 4 others (1974) Carcinoembryonic antigen (CEA) assay. *Hum. Pathol.*, **5**, 139.

KHOO, S. K., DAUNTER, B. & MACKAY, E. (1979*a*) Carcinoembryonic antigen and $\beta_2$ microglobulin as serum tumor markers in women with genital cancer. *Int. J. Gynaecol. Obstet.*, **16**, 388.

KHOO, S. K., HILL, R. & MACKAY, E. V. (1977) Detection of carcinoembryonic antigen and alphafetoprotein in serum and ascitic fluid from patients with ovarian cancer. *Aust. N.Z. J. Obstet. Gynaecol.*, **17**, 94.

KHOO, S. K., WHITAKER, S. V., JONES, I. S. C. & MACKAY, E. V. (1979*b*) Carcinoembryonic antigen in patients with residual ovarian cancer. *Gynecol. Oncol.*, **7**, 288.

LIN, C. W., ORCUTT, M. L. & STOLBACH, L. L. (1975) Elevation of histaminase and its concurrence with Regan isoenzyme in ovarian cancer. *Cancer Res.*, **35**, 2762.

RUTANEN, E. M. & SEPPALA, M. (1978) Carcinoembryonic antigen in malignant and nonmalignant gynecologic tumors. *Cancer*, **42**, 581.

SAMAAN, N. A., SMITH, J. P., RUTLEDGE, F. N. & SCHULTZ, P. N. (1976) The significance of measurement of placental lactogen, human chorionic gonadotropin and carcinoembryonic antigen in patients with ovarian carcinoma. *Am. J. Obstet. Gynecol.*, **126**, 186.

SARJADI, S., DAUNTER, B., MACKAY, E., MAGON, H. & KHOO, S. K. (1980) A multiparametric approach to tumor markers detectable in serum in patients with carcinoma of the ovary or uterine cervix. *Gynecol. Oncol.*, **10**, 113.

SEPPALA, M., PIHKO, H. & ROUSLAHTI, E. (1975) Carcinoembryonic antigen and alphafetoprotein in malignant tumours of the female genital tract. *Cancer*, **35**, 1377.

STONE, M., BAGSHAWE, K. D., KARDANA, A., SEARLE, F. & DENT, J. (1977) Beta-human chorionic gonadotrophin and carcinoembryonic antigen in the management of ovarian carcinoma. *Br. J. Obstet. Gynaecol.*, **84**, 375.

VAN NAGELL, J. R., MEEKER, W. R., PARKER, J. C. & HARRALSON, J. D. (1975) Carcinoembryonic antigen in patients with gynaecologic malignancies. *Cancer*, **35**, 1372.

VECCHIO, T. J. (1966) Predictive value of a single diagnostic test in unselected populations. *N. Engl. J. Med.*, **274**, 1171.